# Inhibition of Cerebellar Dentate Nucleus‐Zona Incerta Glutamatergic Circuit Improves Sleep Disturbances in Chronic Temporal Lobe Epilepsy Model

**DOI:** 10.1002/cns.71207

**Published:** 2026-10-13

**Authors:** Liang Xia, Xuan Wang, Yingchi Zhang, Jianmin Hao, Dan Mi, Changgeng Song, Wen Jiang

**Affiliations:** ^1^ Department of Neurology Xijing Hospital, Air Force Medical University Xi'an China; ^2^ Department of Neurology The First Naval Force Hospital of Southern Theatre Command Zhanjiang China

**Keywords:** cerebellar dentate nucleus, sleep disturbance, temporal lobe epilepsy, zona incerta

## Abstract

**Background:**

Patients with chronic temporal lobe epilepsy (TLE) frequently experience various sleep disorders, characterized by reduced sleep duration, diminished sleep stages, and disrupted sleep patterns. Recent research has identified a population of GABAergic neurons in the ventral zona incerta (ZI) that is instrumental in promoting sleep, along with a neural pathway that connects the cerebellar dentate nucleus (CDN) to the ZI. However, the precise role of this circuit in regulating sleep patterns remains inadequately understood.

**Methods:**

This study employed anatomical, chemogenetic, optogenetic, and immunofluorescence staining techniques to investigate the potential of the CDN‐ZI circuit in ameliorating sleep disturbances associated with TLE.

**Results:**

Our findings indicate that pilocarpine‐treated mice exhibit significant sleep fragmentation and reduced sleep duration, particularly during non‐rapid eye movement sleep. By employing optogenetic and chemogenetic methods to target CDN glutamatergic neurons, we observed a decrease in sleep fragmentation, an increase in overall sleep duration, and an enhancement in sleep quality in pilocarpine‐treated mice. The inhibition of the CDN‐ZI glutamatergic circuit through optogenetic and chemogenetic methods resulted in substantial improvements in sleep disturbances following TLE.

**Conclusions:**

Targeting the CDN‐ZI glutamatergic circuit represents a promising therapeutic strategy for addressing sleep disorders resulting from chronic TLE.

## Graphical abstract

Mice with TLE exhibit significant sleep fragmentation and reduced sleep duration, particularly during non‐rapid eye movement sleep. By employing optogenetic and chemogenetic methods to target CDN glutamatergic neurons, we observed a decrease in sleep fragmentation, an increase in overall sleep duration, and an enhancement in sleep quality among TLE mice. The inhibition of the CDN–ZI glutamatergic circuit resulted in substantial improvements in sleep disturbances following TLE.

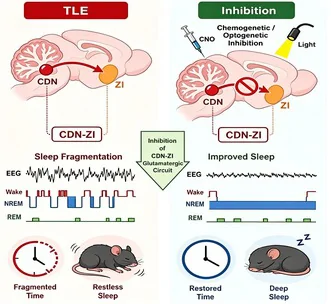

## 1 Introduction

Sleep disturbances frequently occur in patients with epilepsy, particularly in chronic temporal lobe epilepsy (TLE), with bidirectional relationships exacerbating both conditions [1, 2, 3, 4, 5]. Epilepsy and its treatments can profoundly disrupt normal sleep architecture, which in turn may negatively impact seizure control [6]. Sleep disorders in TLE are characterized by shorter night sleep time, reduced or absent sleep phases, and fragmented sleep [3, 4]. Beyond compromising quality of life, such disturbances are significant contributors to cognitive dysfunction, mood disorders, and potentially increased seizure frequency [7]. This vicious cycle poses a substantial challenge to disease management. Despite the well‐recognized clinical significance, effective targeted interventions remain limited, underscoring an urgent need for novel therapeutic strategies.

The cerebellum has increasingly been implicated in a range of disorders associated with sleep disturbances [8, 9, 10]. Recent evidence points to robust interactions between the hippocampus and cerebellum during both non‐rapid eye movement (NREM) and rapid eye movement (REM) sleep [11]. Furthermore, the cerebellum receives modulatory inputs from multiple neuromodulatory systems that are crucial for arousal and sleep–wake regulation, including histaminergic inputs derived from the tuberomammillary nucleus [12], serotonergic signals from the raphe nuclei [13], noradrenergic inputs from the locus coeruleus [14], and orexin/hypocretin inputs from the posterior lateral hypothalamus [15]. Critically, all cerebellar output signals must pass through the deep cerebellar nuclei [16]. Among these, the cerebellar dentate nucleus (CDN)–the largest deep cerebellar nucleus in humans [16, 17], is predominantly composed of glutamatergic projection neurons, which constitute over 80% of its neuronal population [17]. This unique position and composition make the CDN a potentially pivotal neuromodulatory hub for regulating sleep and treating sleep disorders.

The zona incerta (ZI), situated in the hypothalamic area of the thalamus, primarily consists of inhibitory neurons, particularly those that are gamma‐aminobutyric acid (GABA)ergic [18]. Accumulating evidence has established the ZI as a key node in sleep regulation [19, 20, 21, 22]. Specifically, a population of LHX6‐expressing GABAergic neurons in the ventral ZI has been shown to actively promote sleep; selective activation of these neurons prolongs sleep duration, whereas their inhibition suppresses sleep [21]. The integration of sensory and motor signals is a crucial function of the ZI, which dispatches inhibitory GABAergic neurons to different regions of the brain [23]. Notably, the ZI is an established projection area downstream from the glutamatergic neurons from the CDN [21, 24]. This anatomical and functional relationship led us to hypothesize that the glutamatergic circuit connecting the CDN and the ZI may play a critical role in the sleep disruptions seen in chronic TLE. Our previous clinical study focusing on the cerebellum has demonstrated that cerebellar transcranial magnetic stimulation can reduce seizures in patients with refractory temporal lobe epilepsy [25].

In the present study, we employed a combination of anatomical tracing, chemogenetic, optogenetic, and immunofluorescence techniques to systematically investigate the role of the CDN‐ZI glutamatergic pathway in modulating sleep disorders in a mouse model of TLE. Our findings provide direct evidence that targeted inhibition of this circuit significantly alleviates sleep fragmentation and improves sleep quality, establishing the CDN‐ZI glutamatergic pathway as a promising therapeutic target for sleep disorders associated with chronic TLE.

## 2 Materials and Methods

### 2.1 Animals

In this study, *C57BL/6J* adult male mice aged 8–10 weeks were acquired from the Air Force Medical University Laboratory Animal Center. Mice were kept in a controlled environment with a 12‐h light/dark cycle at a temperature of 22°C–25°C and had free access to food and water. All procedures carried out on the animals were in accordance with the guidelines set by the Animal Use and Laboratory Animal Management Ethics Committee of Air Force Medical University and were approved by the Ethics Committee of Xijing Hospital (approval number: XJ‐20231018‐F1).

### 2.2 Stereotaxic Injections

In order to induce anesthesia, mice were given an intraperitoneal injection of pentobarbital sodium at a dosage of 40 mg/kg of body weight. Following this, the animals were positioned on a stereotaxic apparatus (RWD Life Science in Shenzhen, China) and received virus injection via a glass micropipette. Following the injection, the needle remained in position for an additional 10 min before removal.

The coordinates for injection were as follows, according to the Allen brain atlas: CDN: AP, −5.9 mm; ML, 2.3 mm; DV, −3.5 mm. ZI: AP, −1 mm; ML, 0.7 mm; DV, −4.67 mm. The virus was procured from BrainVTA Co. Ltd., Wuhan, China.

Chemogenetic manipulation of the CDN involved the injection of either AAV2/9‐CaMKIIα‐hM4D(Gi)‐mCherry‐WPRE (150 nL) or AAV2/9‐CaMKIIα‐mCherry‐WPRE (150 nL) into the bilateral CDN of C57BL/6J mice for the specific manipulation of CDN glutamatergic neurons.

For optogenetic manipulation of the CDN or CDN‐ZI glutamatergic circuit, AAV2/9‐CaMKIIα‐eNpHR3.0‐EYFP‐WPRE (150 nL), or AAV2/9‐CaMKIIα‐EYFP‐WPRE (150 nL) was injected into the bilateral CDN of C57BL/6J mice. Optic fibers were then implanted above the CDN or ZI 3 weeks later.

To specifically inhibit the CDN‐ZI glutamatergic circuit, AAV2/R‐hSyn‐Cre‐retro (150 nL) was injected into the ZI and AAV2/9‐CaMKIIα‐DIO‐hM4D(Gi)‐mCherry‐WPRE (150 nL) was injected into the bilateral CDN. AAV2/9‐CaMKIIα‐DIO‐mCherry‐WPRE pA (150 nL) was injected into the CDN control.

To explore neural projections downstream of the CDN, AAV2/2‐CaMKIIα‐EYFP (150 nL) was stereotaxically injected into the unilateral CDN. Mice recovered for 4 weeks after injection.

### 2.3 Optogenetic Manipulation

For optogenetic inhibition of the CDN or CDN‐ZI glutamatergic circuit, a 589 nm laser (10 mW, 30 s‐on, 30 s‐off) was applied as detailed in a previous study [26]. Mice that had fiber optic ferrules implanted in either the CDN or ZI were subjected to photostimulation by securing the ferrule to a fiber optic cable equipped with a rotary joint (Doric Lenses), which connected to a fiber‐coupled 589 nm yellow laser (BOGS‐1, Beijing Bio‐Gold Biotech Co. Ltd). The laser power emitted from the fiber optic cable was measured using a photometer (Thorlabs) and adjusted to around 10 mW/mm^2^ to account for a power drop of 10%–20% at the ferrule‐cable junction. During the sleep assessment, each mouse was subjected to stimulation for a duration of 30 min from 8:00 AM to 8:30 AM for a continuous period of 7 days. The fiber implantation site and viral expression were verified using immunofluorescent staining. Data from mice, where the locations of injection or implantation were histologically confirmed to have deviated from the target brain areas, were omitted from subsequent analysis.

### 2.4 Chemogenetic Manipulation

For chemogenetic inhibition of CDN or CDN‐ZI circuit, clozapine N‐oxide dihydrochloride (CNO, 1 mg/kg, Sigma) or 50 μL saline were administered by intraperitoneal injection 30 min before behavioral tests as described before [27]. Chemogenetic manipulation lasted for 7 days. Moreover, to eliminate the chance of CNO influencing the experimental outcomes, control groups were established using hM4Di negative mice that received CNO administration.

### 2.5 Preparation of Lithium‐Pilocarpine Epilepsy Model

The lithium‐pilocarpine epilepsy mouse model was established based on our previous experimental methods [28, 29]. The lithium‐pilocarpine‐treated mice are hereafter referred to as pilocarpine‐treated mice. Each mouse was given an intraperitoneal injection of 425 mg/kg lithium chloride, then an intraperitoneal injection of 100 mg/kg pilocarpine 18–22 h later, in order to trigger an epileptic state. Methylscopolamine (1 mg/kg) was administered subcutaneously 30 min prior to the administration of pilocarpine to minimize peripheral responses. Electrodes were implanted in mice 14 days after pilocarpine injection, and video‐EEG monitoring was conducted on mice 4 weeks after pilocarpine injection. The severity of seizure was behaviorally scored based on video‐electroencephalography recordings by Racine Scale, and categorized as either localized (Racine Scale 1–2) or generalized (Racine Scale 3–6). Epileptic activity persisted for 30 min after successful induction of seizures (intensity≥Racine Level 3 without subsequent return to normal behavior), followed by an intraperitoneal injection of 10 mg/kg diazepam. The control group (Con group) received normal saline instead of pilocarpine, with the other reagents used consistent with that of the model mice.

### 2.6 Video‐EEG–EMG Recordings and Sleep Analysis

Video‐EEG recordings and subsequent analyses were conducted on C57BL/6J mice (male, 8–10 weeks of age, 20–25 g), following electrode implantation, utilizing a video‐EEG monitoring system (Neurostudio System, Jiangsu Brain Medical Technology Co. Ltd., China), according to our previous study [30]. Mice were initially anesthetized using isoflurane and secured on a stereotaxic apparatus (RWD, Shenzhen, China). The head mount (Kedou Brain Computer Technology Co. Ltd., China) was positioned anterior to the bregma by 1.5 mm and secured using stainless steel screws and dental cement. For electromyography (EMG) recording, leads were inserted into the trapezius muscle. After a recovery period of at least one week, each mouse was placed individually in transparent barrels located inside a soundproof recording chamber. These mice experienced a 12‐h light/12‐h dark cycle, with lights turning on at 7:00 AM. Following this, a 7‐day video‐EEG monitoring session was performed on freely moving mice within the soundproof chamber. The EEG and EMG signals originating from the cortex were both amplified and filtered. They were passed through a high‐pass filter set above 0.5 Hz and a band‐pass filter ranging from 0.5 to 45 Hz, then digitized at a resolution of 1000 Hz through a tethered data acquisition system, and synchronized with the infrared video footage. The sleep–wake states were assessed every 4 s and classified into wakefulness, NREM, or REM sleep using established criteria from previous research [31, 32]. Specifically, wakefulness was identified by desynchronized, low‐amplitude EEG patterns and increased EMG activity. NREM sleep was characterized by synchronized, high‐amplitude, low‐frequency (0.1–4 Hz, delta) EEG activity, with a notable reduction in EMG activity compared to wakefulness. REM sleep was defined by the presence of consecutive theta (5–10 Hz) oscillations and a further decrease in EMG activity relative to the preceding NREM sleep. The power for the δ (0.5–4 Hz), θ (5–8 Hz), and α (9–14 Hz) frequency bands, in addition to the EMG/EEG activity signal, was computed and analyzed. The vigilance states of the pilocarpine‐treated mice were then assessed using the artificial intelligence‐driven software Lunion Stage, which was created by LunionData [33]. Short awakenings were characterized as incidents where there was a reduction of at least 50% in EEG strength for 5–15 s in the 1–4 Hz frequency range while in NREM sleep according to our previous study [30]. The Video‐EEG/EMG information was examined after the fact utilizing Lunion Stage software by analysts who were unaware of the categories. The breakdown of various levels of vigilance states was evaluated [34].

Next, using data from EEG/EMG monitoring, we computed a series of parameters including total sleep duration, awakenings per hour, bouts per hour, awake time duration per hour, NREM sleep duration per hour, and REM sleep duration per hour. We visually inspected the EEG to prevent the EEG during a seizure from being mistaken for the waking phase.

### 2.7 Immunohistochemistry

The animals were anesthetized using 100 mg/kg of pentobarbital sodium administered intraperitoneally and then perfused with phosphate‐buffered saline at a pH of 7.4, followed by 4% paraformaldehyde in phosphate buffer (PB) at a pH of 7.4 for fixation. After soaking in 4% paraformaldehyde for 2–4 h, the brains were moved to 30% sucrose in 0.1 M PB until they sank. The brains were then cut into 20 μm transverse sections using a Leica CM1950 freezing microtome and kept in PBS at 4°C until immunostaining. Then the sections were treated with 5% donkey serum for 30 min. After blocking, the sections were transferred to wells and incubated with a primary antibody against CaMKIIα and Fos for 24 h at 4°C in a blocking solution. Subsequently, the sections were washed with 0.4% Triton in PBS and incubated for 3 h at room temperature with secondary antibodies, either Alexa Fluor 488 or Alexa Fluor 594. Fluorescence microscopic images of the neurons were captured using an Olympus VS200 fluorescence microscope from Japan.

### 2.8 Statistical Analysis

Data are presented as mean ± SEM, unless specified otherwise. Prior to analysis, all data were subjected to the Kolmogorov–Smirnov test for normality. For comparisons between two independent groups, an unpaired Student's *t*‐test was used when data were normally distributed; otherwise, a Mann–Whitney U test was used. For paired comparisons, such as before and after intervention in the same mice, a paired Student's *t*‐test was used. For comparing three or more independent groups, one‐way ANOVA followed by Dunn's multiple comparisons test was employed. Correlation analysis was performed using Pearson correlation analysis. A statistically significant *p* value was considered to be less than 0.05. The statistical analyses were carried out using GraphPad Prism 9 software (GraphPad, San Diego, CA, USA).

## 3 Results

### 3.1 Pilocarpine‐Treated Mice Exhibited Significant Sleep Disorders

Previous research has shown that the lithium‐pilocarpine rat model is appropriate for investigating sleep disturbances in epilepsy, and it has been found that the integrity of NREM sleep is disrupted [35]. To assess the impact of pilocarpine on the sleep wake cycle of mice, we conducted continuous EEG/EMG analysis on the mice 4 weeks after pilocarpine modeling and compared it with a control group of mice. A total of 72 mice that survived after reaching status epilepticus following pilocarpine treatment were included in the study, resulting in a successful induction rate of 73.46% (72/98). During the acute phase of epilepsy, the model group exhibited reduced food and water intake, slowed weight gain, irritability, increased aggression, and intermittent convulsive seizures lasting from several seconds to 1 min. These symptoms gradually diminished 3–5 days after the onset of epileptogenesis. Around the third week post‐epileptogenesis, spontaneous recurrent seizures (SRS) began to occur, characterized by mechanical head nodding, chewing, forelimb clonus, rearing, and occasionally generalized tonic–clonic seizures. However, these seizures were brief, lasting approximately 30 s to 1 min each, and were capable of self‐termination. By the 4th week post‐epileptogenesis, spontaneous seizures had stabilized. At this stage, the model group consisted of 72 surviving mice that had experienced status epilepticus, all of which exhibited SRS. The seizure frequency was recorded at 6.85 ± 1.07 times per day, with an average duration of 37.98 ± 7.59 s per episode, demonstrating statistically significant differences compared to the control group (Figure S1A–C). Notably, seizure frequency was significantly higher during the light phase than during the dark phase (Figure S1D), suggesting a potential interaction between epileptiform activity and circadian sleep–wake rhythms. Therefore, we propose that the pathological reorganization of neural circuits induced by the pilocarpine‐induced TLE model contributes to sleep disorders, rather than frequent seizures disrupting sleep patterns.

Following the sleep analysis protocol, we continuously monitored the C57 mice for 7 days. The sleep conditions of each group were analyzed using the Lunion Stage software to determine the duration of wake, NREM sleep, and REM sleep phases. The power spectrum and sleep stage results (Figure 1A,B) revealed that control mice displayed a complete sleep–wake cycle (Figure 1C), whereas pilocarpine‐treated mice experienced numerous short awakenings, which resulted in fragmented NREM sleep (Figure 1D). Clinical and electrophysiological studies have demonstrated that sleep fragmentation, characterized by frequent arousals, reduced sleep efficiency, and disrupted sleep architecture, exacerbates epileptiform activity and seizure burden [36, 37, 38]. To assess whether the sleep fragmentation observed in pilocarpine‐treated mice is associated with epilepsy, we examined the relationship between the frequency of awakenings and both the frequency and duration of SRS. Our results demonstrated a significant correlation between the frequency of awakenings and the frequency of SRS (*r* = 0.46, 95% CI [0.06, 0.73], *n* = 24, *p* < 0.05) (Figure S1E). Furthermore, the frequency of awakenings was directly related to the duration of SRS (*r* = 0.62, 95% CI [0.29, 0.82], *n* = 24, *p* = 0.0012) (Figure S1F). These correlation analyses were performed using the same cohort of pilocarpine‐treated mice (*n* = 24), indicating a direct association between sleep fragmentation and epileptic activity. To assess whether the CDN is activated during chronic seizures, we examined FOS protein expression in the CDN following epileptic episodes. FOS expression was significantly elevated in the CDN of pilocarpine‐treated mice compared to controls (t = 6.223, df = 16, *p* < 0.001) (Figure S1G–H), providing indirect evidence of CDN engagement during seizure activity.

**FIGURE 1:**
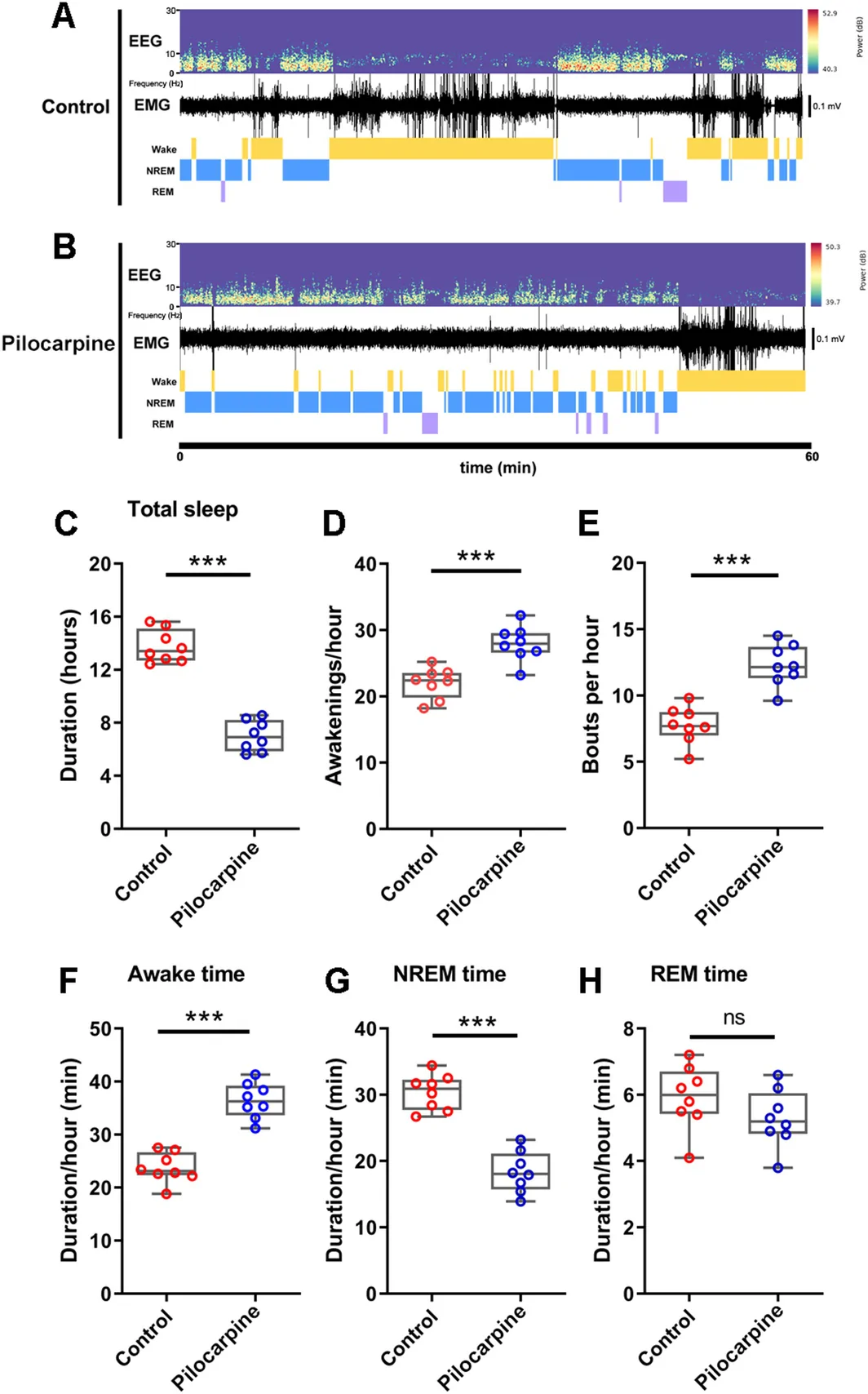
Pilocarpine‐treated mice exhibit significant sleep disturbances. The flow chart illustrates the sleep experiment conducted on control group (A) and pilocarpine group (B), alongside representative EEG power spectrum, electromyography, and sleep stage maps. The flow chart outlines the experiment process for both groups. The sleep stage maps display the variation in power density in EEG power spectra through color intensity, with yellow, blue, and purple indicating Wake, NREM sleep, and the proportion of REM sleep, respectively. The EEG power spectrum is measured in microvolts square per Hertz. The stages represented include Wake, NREM (non‐rapid eye movement sleep), and REM (rapid eye movement sleep). Statistical analysis was conducted on various sleep parameters in the pilocarpine group and control group. These included total sleep duration (C), transient awakening frequency (D), frequency of continuous sleep (E), duration of awakening (F), NREM sleep duration (G), and REM sleep duration (H). *n* = 8 mice per group; The data are presented as mean ± SEM. NREM, non‐rapid eye movement sleep; REM, rapid eye movement sleep; Statistical significance was determined using an Unpaired Student's *t*‐test, **p < 0.01; ***p < 0.001. ns, no significant difference.

Further analysis of sleep‐related indicators in pilocarpine‐treated mice revealed a significant decrease in total sleep time compared with that in control mice (*t* = 11.34, *n* = 16, *p* < 0.0001) (Figure 1C). Furthermore, pilocarpine‐treated mice experienced a higher number of transient awakening events than control mice (*t* = 4.783, *n* = 16, *p =* 0.0003) (Figure 1D), and an increased number of NREM bouts per hour (*t* = 6.117, *n* = 16, *p* < 0.0001) (Figure 1E). Specifically, the awake time of pilocarpine‐treated mice increased significantly compared with that of control mice (*t* = 8.166, *n* = 16, *p <* 0.0001) (Figure 1F, Figure S4A), and pilocarpine‐treated mice showed a decrease in NREM sleep duration (*t* = 8.354, *n* = 16, *p* < 0.0001) (Figure 1G, Figure S4B) but no change in REM sleep duration (*t* = 1.390, *n* = 16, *p =* 0.19) (Figure 1H, Figure S4C). These findings suggest that reduced total sleep duration in pilocarpine‐treated mice is primarily attributable to decreased NREM sleep duration, increased wake time, and an elevated number of short awakenings, indicating sleep fragmentation and compromised sleep quality in pilocarpine‐treated mice.

### 3.2 Chemogenetic Inhibition of the CDN Glutamatergic Neurons Ameliorated Sleep Disorders in Pilocarpine‐Treated Mice

Following the experimental protocol, pilocarpine‐treated mice were successfully modeled and then received stereotaxic injection of AAV2/9‐CaMKIIα‐hM4D(Gi)‐mCherry (hM4D) or AAV2/9‐CaMKIIα‐mCherry (mCherry) into the bilateral CDN (Figure 2A). Simultaneously, EEG/EMG implantation was performed, with continuous monitoring and analysis performed four weeks later. Immunohistochemistry confirmed successful injection of the chemogenetic virus into the CDN region, which is rich in glutamatergic neurons (Figure 2B). EEG/EMG recordings were conducted on pilocarpine‐treated mice treated with either normal saline (Saline group) or 1 mg/kg CNO (CNO group) for seven consecutive days, with sleep data from pilocarpine‐treated mice. The power spectrum and sleep staging results were depicted in Figure 2C,D.

**FIGURE 2:**
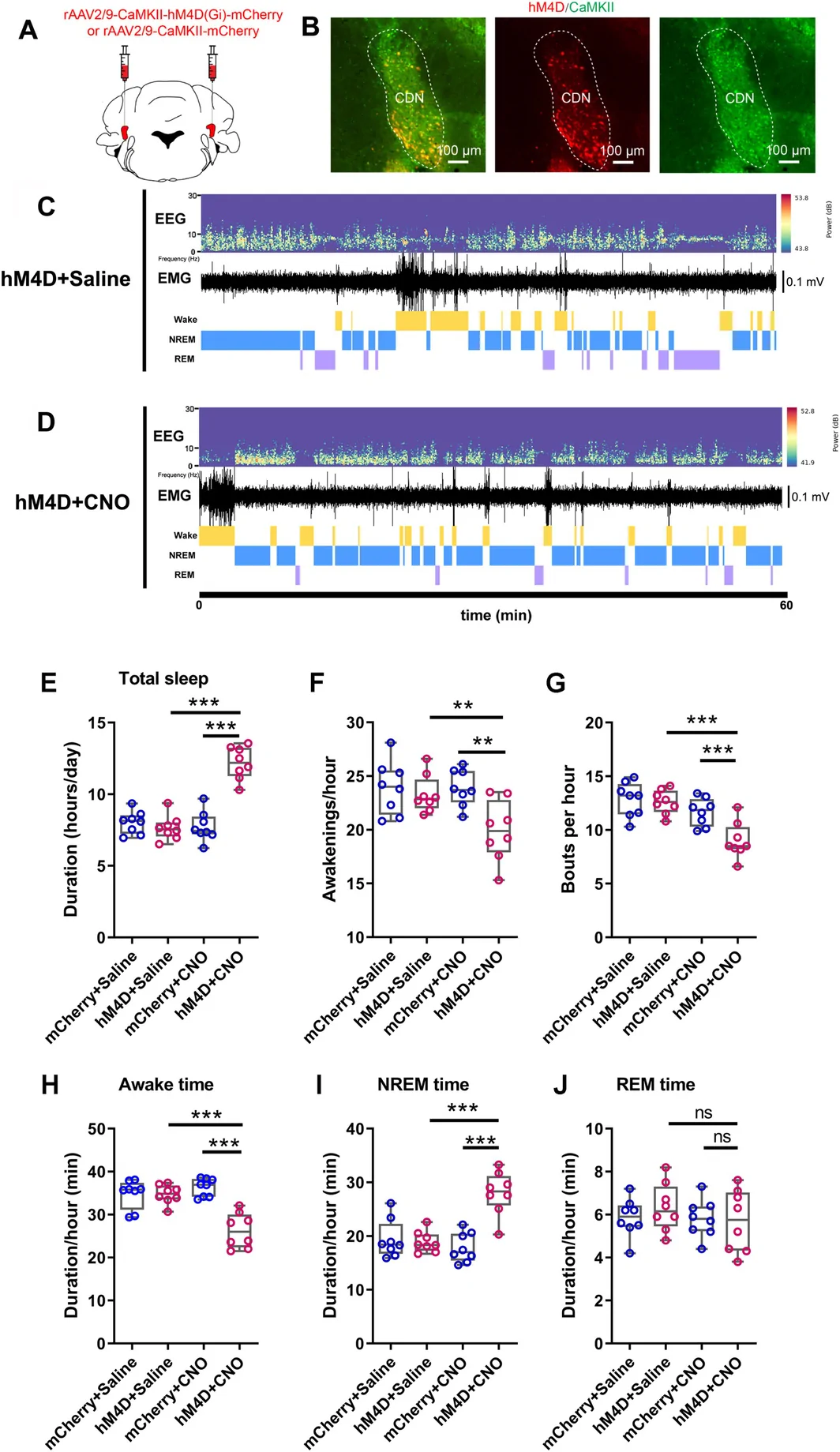
Effects of chemogenetic inhibition of CDN glutamatergic neurons on sleep disorders. (A) Stereotaxic injection of chemogenetic virus AAV2/9‐CaMKIIα‐hM4D(Gi)‐mCherry or control virus AAV2/9‐CaMKIIα‐mCherry in CDN of pilocarpine‐treated mice. (B) Immunofluorescence image of the chemogenetic virus injected into the CDN and the virus colocalized with the CaMKII antibody. Scale bars, 200 μm. (C, D) EEG power spectrum, EMG, and typical sleep stage maps of mice in the hM4D + Saline and hM4D + CNO groups. The variation in the power density in the EEG power spectra is represented by the color intensity. Yellow, blue, and purple at the bottom of the EMG represent the proportions of Wake, NREM sleep, and REM sleep states, respectively. The EEG power spectrum was measured in square microvolts per Hertz. NREM, non‐rapid eye movement; REM, rapid eye movement. The sleep staging results of mice after Saline or CNO treatment were analyzed after chemogenetic inhibition. (E) Effects of chemogenetic inhibition of CDN on total sleep duration after treatment with Saline or CNO. (F) Effects of chemogenetic inhibition of CDN on the frequency of transient awakening after Saline or CNO administration. (G) Effects of chemogenetic inhibition of CDN on the frequency of continuous NREM bouts per hour after Saline or CNO administration. (H) Effects of chemogenetic inhibition of CDN on the duration of awake time after Saline or CNO administration. (I) Effects of chemogenetic inhibition of CDN on the duration of NREM sleep after treatment with Saline or CNO. (J) Effects of chemogenetic inhibition of CDN on the duration of REM sleep after Saline or CNO administration. *n* = 8 mice per group. Statistical significance was determined using the one‐way ANOVA followed by Dunn's multiple comparisons test (**p < 0.01; ***p < 0.001 compared to the hM4D + CNO group). ns, no significant difference.

The hM4D + CNO group exhibited a significant increase in total sleep time compared with those in the hM4D + Saline group (*F*_3,28_ = 41.17, *p* < 0.0001) (Figure 2E). Additionally, the number of awakenings per hour was significantly reduced in the hM4D + CNO group compared with the hM4D + Saline group (*F*_3,28_ = 5.919, *p =* 0.0029) (Figure 2F), while NREM bouts per hour were also significantly reduced in the hM4D + CNO group (*F*_3,28_ = 11.86, *p* < 0.0001) (Figure 2G), indicating an improvement in sleep fragmentation following chemogenetic inhibition of the CDN. Moreover, the duration of the awake period in the hM4D + CNO group was significantly shorter than that in the hM4D + Saline group (*F*_3,28_ = 18.07, *p* < 0.0001) (Figure 2H, Figure S5A). Furthermore, the NREM sleep duration in the hM4D + CNO group was notably longer than that in the hM4D + Saline group (*F*_3,28_ = 17.67, *p* < 0.0001) (Figure 2I, Figure S5B), whereas there was no significant difference in the REM sleep duration between the four groups (*F*_3,28_ = 0.5302, *p =* 0.66) (Figure 2J, Figure S5C). Thus, chemogenetic inhibition of CDN glutamatergic neurons effectively improves sleep continuity and increases NREM sleep duration in pilocarpine‐treated mice.

Next, we investigated whether selective chemogenetic inhibition of glutamatergic neurons in CDN could suppress chronic seizures. In the pilocarpine‐treated mice, we found that chemogenetic inhibition of CDN glutamatergic neurons (CNO group) did not significantly reduce the frequency of convulsive seizures in pilocarpine‐treated mice compared to the Saline group (*t* = 1.157, df = 14, *p* = 0.27) (Figure S2A). Furthermore, there was no significant difference in the duration of single convulsive seizures (*t* = 0.426, df = 14, *p* = 0.68) (Figure S2B). However, following the injection of the control virus for chemogenetic inhibition into the CDN, we found no significant effect on either the frequency of convulsive seizures (*t* = 0.459, df = 14, *p* = 0.65) (Figure S2C) or the duration of single convulsive seizures in pilocarpine‐treated mice (*t* = 0.307, df = 14, *p* = 0.76) (Figure S2D).

To investigate the long‐term effects of chemogenetic inhibition of CDN on sleep, we measured sleep metrics 3 days following the cessation of CDN inhibition. The results indicated that, compared to the hM4D + Saline group, the hM4D + CNO group exhibited a significant increase in total sleep time (*F*_3,28_ = 5.939, *p* = 0.002) (Figure S5D). Additionally, the number of awakening episodes per hour was significantly reduced in the hM4D + CNO group compared to the hM4D + Saline group (*F*_3,28_ = 5.993, *p* = 0.0027) (Figure S5E), and the number of NREM episodes per hour was also significantly lower in the hM4D + CNO group (*F*_3,28_ = 5.667, *p* = 0.0037) (Figure S5F), indicating that sleep fragmentation improved following chemogenetic inhibition of the CDN. Furthermore, the duration of wakefulness episodes in the hM4D + CNO group was significantly shorter than in the hM4D + Saline group (*F*_3,28_ = 6.319, *p* = 0.0021) (Figure S5G). Additionally, the duration of NREM sleep in the hM4D + CNO group was significantly longer than that in the hM4D + Saline group (*F*_3,28_ = 6.475, *p* = 0.0018) (Figure S5H), while no significant differences were observed in REM duration among the four groups (*F*_3,28_ = 0.4231, *p* = 0.73) (Figure S5I). The sleep‐promoting effects of CDN inhibition persisted for at least 3 days after cessation, as indicated by sustained improvements in total sleep time, NREM sleep quality, and reduced fragmentation.

### 3.3 Sleep Disorders in Pilocarpine‐Treated Mice Were Improved Through Optogenetic Inhibition of CDN Glutamatergic Neurons

Our study demonstrated that chemogenetic inhibition of the CDN can alleviate sleep disorders in pilocarpine‐treated mice. Following the experimental protocol, we conducted stereotaxic injections of optogenetic inhibitory viruses AAV2/9‐CaMKIIα‐eNpHR3.0‐EYFP (eNpHR) or control virus AAV2/9‐CaMKIIα‐EYFP (EYFP) into the bilateral CDN region, along with EEG/EMG implantation (Figure 3A). Four weeks after injection, continuous EEG/EMG monitoring analysis was performed. Immunohistochemistry confirmed the successful viral injection into the CDN region and the virus binds well to the glutamate neurons (Figure 3B). EEG/EMG recordings were conducted on pilocarpine‐treated mice under two conditions: without yellow light stimulation (No Light group) and with yellow light stimulation (Yellow Light group) for seven consecutive days. Sleep patterns were analyzed using Lunion Stage software, focusing on the wake, NREM sleep, and REM sleep phases. The eNpHR + No Light group showed NREM sleep fragmentation and prolonged night awakening time (Figure 3C,D). Pilocarpine‐treated mice exposed to eNpHR + Yellow light exhibited a notable increase in total sleep time (*F*_3,28_ = 40.90, *p* < 0.0001) (Figure 3E) along with a decrease in awakenings/hour (*F*_3,28_ = 14.95, *p* < 0.0001) (Figure 3F) and NREM bouts per hour (*F*_3,28_ = 6.062, *p =* 0.0026) (Figure 3G) compared with the eNpHR + No Light group. This indicated an amelioration of sleep fragmentation in pilocarpine‐treated mice following optogenetic inhibition of the CDN. Furthermore, the eNpHR + Yellow Light group showed a significant reduction in awake duration compared with the eNpHR + No Light group (*F*_3,28_ = 55.03, *p* < 0.0001) (Figure 3H, Figure S6A), and NREM sleep duration was significantly longer than that in eNpHR + No Light group (*F*_3,28_ = 64.98, *p* < 0.0001) (Figure 3I). Conversely, there was no significant difference in REM sleep duration between the four groups (*F*_3,28_ = 0.05, *p =* 0.98) (Figure 3J, Figure S6C). These findings suggest that optogenetic inhibition of the CDN glutamatergic neurons not only increases total sleep time and improves NREM sleep quality, but also reduces awake time and short awakenings, ultimately alleviating sleep fragmentation in pilocarpine‐treated mice.

**FIGURE 3:**
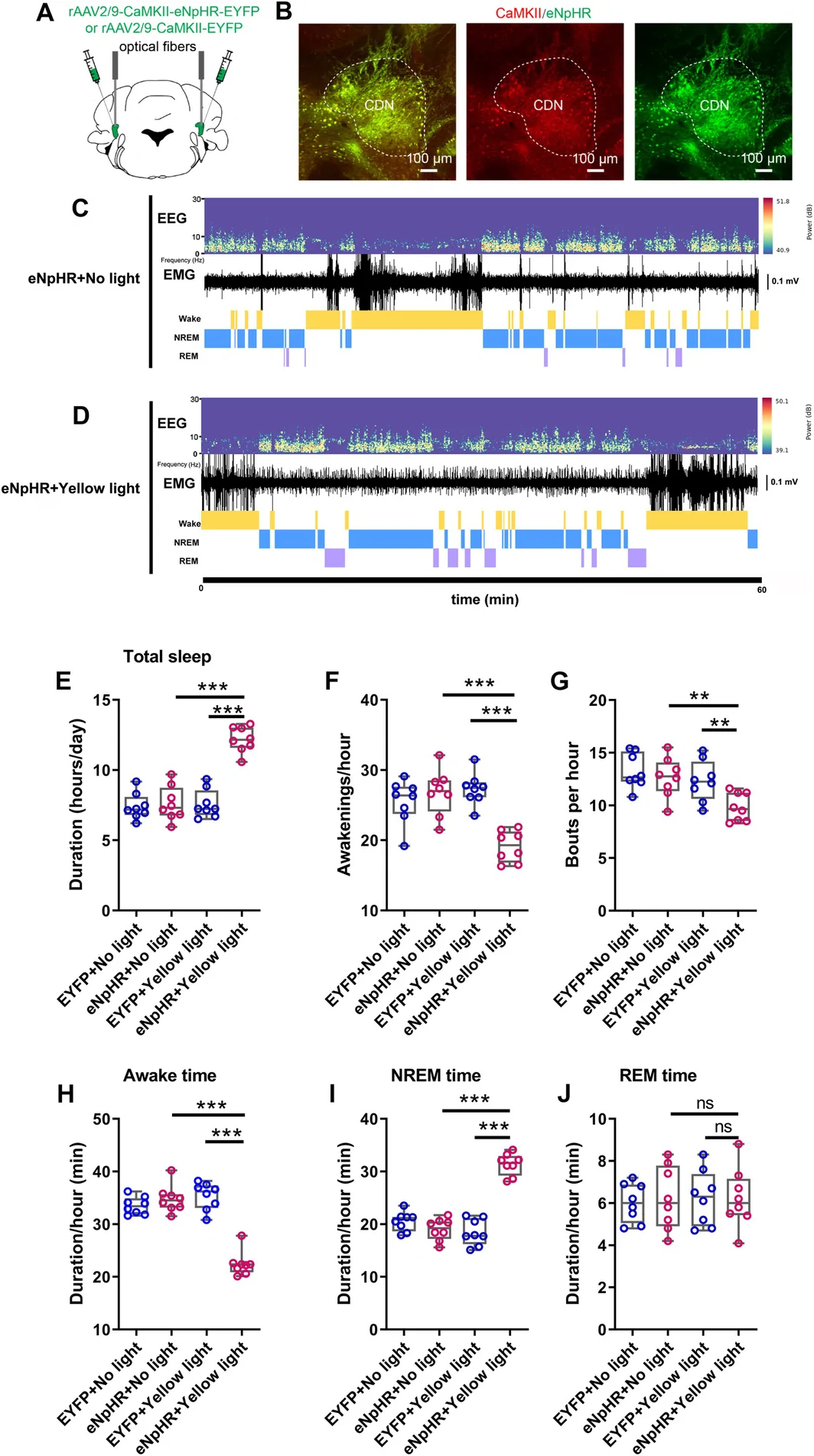
Effects of optogenetic inhibition of CDN glutamatergic neurons on sleep disorders. (A) Stereotaxic injection of the optogenetic virus AAV2/9‐CaMKIIα‐eNpHR3.0‐EYFP or control virus AAV2/9‐CaMKIIα‐EYFP was performed in the CDN of pilocarpine‐treated mice. (B) Immunofluorescence images show the juxtaposed optical fibers injected into the CDN and the virus colocalized with the CaMKII antibody. (C, D) The EEG power spectrum, electromyography (EMG), and sleep stage map of mice in the eNpHR + No light group (C) and eNpHR + Yellow light group (D) are presented. The color intensity in the EEG power spectra indicates the variation in power density. Yellow, blue, and purple at the bottom of the EMG represent the proportion of Wake, NREM sleep, and REM sleep, respectively. The EEG power spectrum is measured in square microvolts per hertz. (E) Effects of optogenetic inhibition of CDN on total sleep duration between the EYFP + No light, eNpHR + No light, EYFP + Yellow light, and eNpHR + Yellow light groups. (F) Effects of optogenetic inhibition of CDN on transient awakening frequency (times/h) during the night between each group. (G) Effects of optogenetic inhibition of CDN on the frequency of continuous sleep in the night between each group. (H) Effects of optogenetic inhibition of CDN on the duration of arousal at night between each group. (I) Effects of optogenetic inhibition of CDN on NREM sleep duration at night between each group. (J) Effects of optogenetic inhibition of CDN on the duration of REM sleep at night between each group. *n* = 8 mice per group. The data are presented as mean ± SEM. NREM, non‐rapid eye movement phase of sleep; REM, rapid eye movement sleep. Statistical significance was determined using one‐way ANOVA followed by Dunn's multiple comparisons test (*p < 0.05, **p < 0.01, ***p < 0.001, compared with eNpHR + Yellow light group; ns, no statistical difference).

To investigate the long‐term effects of optogenetic inhibition of CDN glutamatergic neurons on sleep, we measured sleep metrics 3 days following the cessation of CDN inhibition. Pilocarpine‐treated mice exposed to eNpHR + Yellow light exhibited a notable increase in total sleep time (*F*_3,28_ = 4.811, *p* = 0.008) (Figure S6D) along with a decrease in awakenings/hour (*F*_3,28_ = 4.658, *p* = 0.0092) (Figure S6E) and NREM bouts per hour (*F*_3,28_ = 3.508, *p* = 0.0282) (Figure S6F) compared with the eNpHR + No Light group. This indicated an amelioration of sleep fragmentation in pilocarpine‐treated mice following optogenetic inhibition of the CDN. Furthermore, the eNpHR + Yellow Light group showed a significant reduction in awake duration compared with the eNpHR + No Light group (*F*_3,28_ = 4.052, *p* = 0.0164) (Figure S6G), and NREM sleep duration was significantly longer than that in the eNpHR + No Light group (*F*_3,28_ = 5.529, *p* = 0.0041) (Figure S6H). Conversely, there was no significant difference in REM sleep duration between the four groups (*F*_3,28_ = 0.5317, *p* = 0.6643) (Figure S6I). The beneficial effects on sleep architecture were maintained 3 days after optogenetic inhibition ceased.

Next, we investigated whether selective optogenetic inhibition of glutamatergic neurons in CDN could suppress chronic epileptic seizures. In the pilocarpine‐induced TLE mice model, we found that, compared to the No‐Light group, optogenetic inhibition of glutamatergic neurons in the CDN (Light group) did not significantly reduce the frequency of convulsive seizures in pilocarpine‐treated mice (*t* = 1.215, df = 14, *p* = 0.24) (Figure S3A), nor was there a significant difference in the duration of single convulsive seizures (*t* = 0.897, df = 14, *p* = 0.39) (Figure S3B). Additionally, following the injection of the control virus for optogenetic inhibition into the CDN, no significant effects were observed on the frequency of convulsive seizures (*t* = 0.837, df = 14, *p* = 0.42) (Figure S3C) or the duration of single convulsive seizures in pilocarpine‐treated mice (*t* = 1.873, df = 14, *p* = 0.08) (Figure S3D).

### 3.4 The Downstream Projection Area of the CDN and the Verification of the CDN‐ZI Glutamatergic Circuit

Previous study showed that there is a CDN‐ZI glutamatergic circuit that may regulate the anxiety‐like behaviors [24], we aim to investigate the downstream projection area of the CDN and the verification of the CDN‐ZI glutamatergic neural circuit. We utilized the anterograde tracer method by injecting the anterograde tracer virus AAV2/2‐CaMKIIα‐EYFP into the CDN. This AAV selectively infects excitatory neurons in the brain [26]. Following one month of virus expression, mouse brains were cut into 20 μm sections and aligned according to the Allen mouse brain atlas; the labeled structures in downstream regions are axonal terminals. We observed a significant presence of green fluorescent AAV in the CDN area under a low‐power microscope (Figure 4A, *n* = 6 mice). Subsequent whole‐brain fluorescence scanning using the VS200 system at various cerebellar levels demonstrated the high efficiency of the AAV as an anterograde tracer for studying the neural circuitry originating from the CDN. The green fluorescently labeled neurons projecting anterogradely with the AAV2/2‐CaMKIIα‐EYFP virus were observed in different brain planes ranging from Bregma‐1.8 mm to Bregma‐3.0 mm (Figure 4B–F,N). Notably, regions such as the ventral posterolateral thalamic nucleus (VPL), ventromedial thalamic nucleus (VM), ventral posteromedial thalamic nucleus (VPM), ZI, prerubral field (PR), and retroparafascicular nucleus (RPF) exhibited dense distributions of labeled axon terminals (Figure 4C–K), particularly within the ZI area (Figure 4B,D,L). We found a significant number of CDN recipient neurons located in the ZI; approximately 26.5% of glutamatergic CDN neurons projected to ZI, and nearly all of these projecting neurons were identified as glutamatergic (Figure 4M). These results confirmed the existence of CDN‐ZI glutamatergic circuit.

**FIGURE 4:**
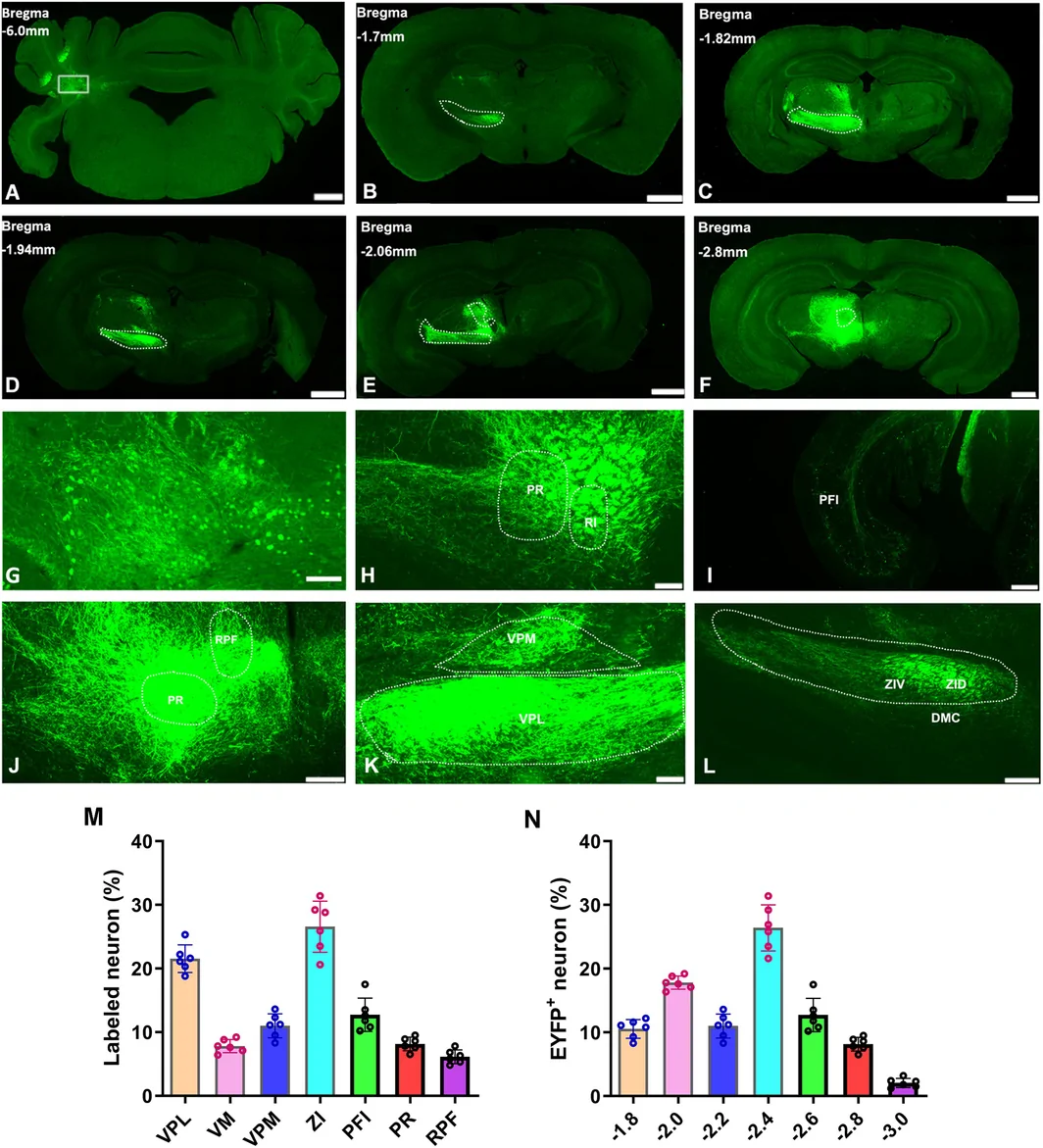
The AAV virus anterograde tracer method was used to demonstrate the downstream neural projection of the CDN to the ZI. (A) Fluorescence results of AAV retrograde tracer AAV2/2‐CaMKIIα‐EYFP following stereotaxic injection into the CDN. (B) Displays the image of the region projected by the AAV virus to the zona incerta (ZI) region of the thalamus. (C, D) Show the images projected by the AAV virus into the ZI, reticular thalamic nucleus (RPF), and parafascicular nucleus (PF) regions of the thalamus. (E, F) depict the images projected by the AAV virus to the RPF and PF regions of the thalamus. The ventroposterolateral nucleus of the thalamus (VPL) is labeled. Scale bars, 1 mm (A–F). (G) Shows a magnified view of the AAV virus in the CDN region of Figure A. (H) the AAV virus projection is observed in the PR area. (I) Shows the projection to the PFI region of the cerebellar cortex. (J) Provides a magnified view of the AAV virus projection in the PR and RPF regions. (K) Offers a magnified view at the projection to the VPL and VPM regions. (L) Illustrates the projection by AAV virus to the ZIV and ZID regions. Abbreviations: VPL ventral posterolateral thalamus nucleus, VPM ventral posteromedial thalamic nucleus, ZIV zona incerta, ventral part, ZID zona incerta, dorsal part, PR prerubral region, and PFI paraflocculus. Scale bars, 200 μm (G, I, J, L), 100 μm (H, K). (M) Projection rates were determined by calculating the ratio of traced neurons located in the ZI that originated from the starter neuron in the CDN. *n* = 6 mice per group. (N) The quantity of glutamatergic neurons in the ZI that originated from the CDN was measured along the anteroposterior (AP) axis. *n* = 6 mice per group.

### 3.5 Chemogenetic Inhibition of the CDN‐ZI Glutamatergic Circuit Improved Sleep Disorders Following TLE

To investigate the role of the CDN‐ZI glutamatergic circuit in sleep regulation, we used chemogenetic techniques to inhibit the CDN‐ZI glutamatergic circuit and assessed its impact on sleep disturbances after TLE. Following a standardized experimental protocol for pilocarpine‐treated mice, we conducted stereotaxic injections of the chemogenetic inhibitory virus AAV‐CaMKIIα‐DIO‐hM4D(Gi)‐mCherry (hM4D) or AAV‐CaMKIIα‐DIO‐mCherry (mCherry) into the bilateral CDN and retro‐rAAV‐hSyn‐Cre virus into the ZI (Figure 5A). Immunohistochemistry confirmed the presence of the CDN‐ZI glutamatergic circuit after injection of the chemogenetic virus (Figure 5B). The results indicated that mice in the hM4D + Saline group exhibited significant NREM sleep fragmentation and extended night awake duration, whereas mice in the hM4D + CNO group displayed enhanced sleep quality, increased NREM sleep duration, and reduced wake time following chemogenetic inhibition of glutamatergic neurons in the CDN (Figure 5C,D).

**FIGURE 5:**
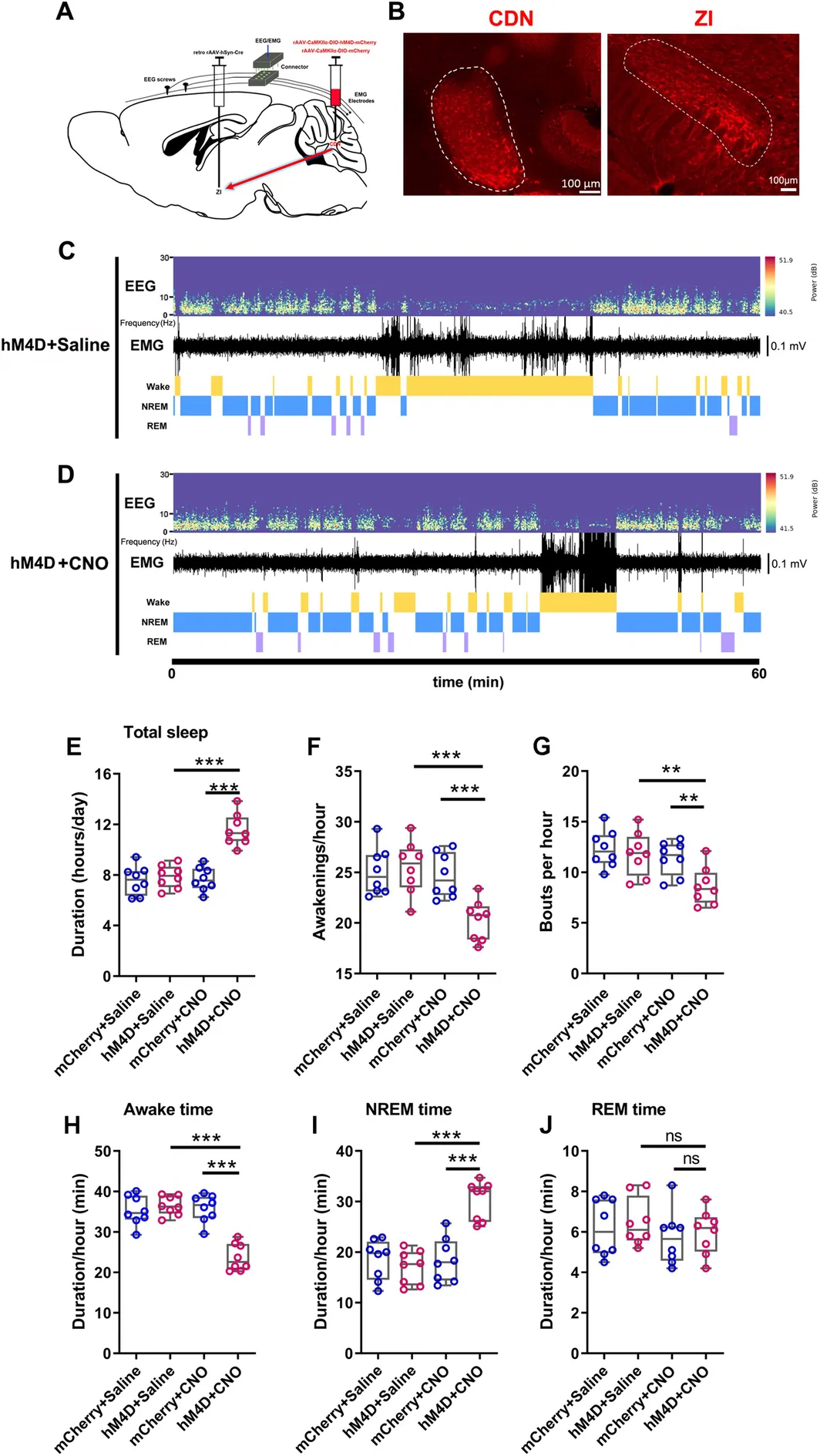
Effect of chemogenetic inhibition of CDN‐ZI glutamatergic circuit on sleep disturbances after TLE. (A) Pilocarpine‐treated mice were stereotaxically injected with either chemogenetic virus AAV2/9‐CaMKIIα‐DIO‐hM4D(Gi)‐mCherry (hM4D) or control virus AAV2/9‐CaMKIIα‐DIO‐mCherry (mCherry) in the CDN, while the retro rAAV‐hSyn‐Cre virus was injected into the ZI region. (B) Shows an immunofluorescence map of the chemogenetic virus injected into the CDN. Scale bars, 100 μm. (C, D) Display EEG power spectrum, EMG, and sleep stage typical maps of mice in the hM4D + Saline group and hM4D + CNO group. The color intensity in the EEG power spectra indicates the variation in power density, while the colors at the bottom of the EMG represent Wake (yellow), NREM sleep (blue), and REM sleep (purple). The EEG power spectrum is measured in square microvolts per Hertz. Sleep staging results of mice in the mCherry + Saline group, hM4D + Saline group, mCherry + CNO group and hM4D + CNO group were analyzed after chemogenetic inhibition of the CDN‐ZI glutamatergic circuit. Statistical analysis included total sleep duration at night (E), transient awakenings per hour (F), bouts per hour (G), awake duration per hour (H), NREM sleep duration per hour (I), and REM sleep duration per hour (J). *n* = 8 mice per group; The data are presented as mean ± SEM; Statistical significance was determined using one‐way ANOVA followed by Dunn's multiple comparisons test (**p < 0.01, ***p < 0.001, ns, no significant difference). NREM, non‐rapid eye movement sleep; REM, rapid eye movement sleep.

Pilocarpine‐treated mice in the hM4D + CNO group exhibited a significant increase in total sleep time compared with those in the hM4D + Saline group (*F*_3,28_ = 26.21, *p* < 0.0001) (Figure 5E). Conversely, the number of awakenings per hour (*F*_3,28_ = 8.99, *p* = 0.0002) (Figure 5F) and NREM bouts per hour were significantly reduced in the hM4D + CNO group compared with those in the hM4D + Saline group (*F*_3,28_ = 6.10, *p =* 0.0025) (Figure 5G), indicating an improvement in sleep fragmentation in TLE. Furthermore, the duration of awake periods per hour was significantly shorter in the hM4D + CNO group than in the hM4D + Saline group (*F*_3,28_ = 28.60, *p* < 0.0001) (Figure 5H, Figure S7A). Additionally, the NREM sleep duration in the hM4D + CNO group was significantly longer than that in the hM4D + Saline group (*F*_3,28_ = 20.38, *p* < 0.0001) (Figure 5I, Figure S7B), with no significant difference in REM sleep duration between the four groups (*F*_3,28_ = 0.54, *p =* 0.65) (Figure 5J, Figure S7C). These findings suggest that chemogenetic inhibition of the CDN‐ZI glutamatergic circuit leads to increased total sleep time and improved NREM sleep quality in pilocarpine‐treated mice by reducing the awake time and short awakenings.

To investigate the short‐term effects of chemogenetic inhibition of CDN‐ZI glutamatergic circuit on sleep, we measured sleep metrics one day following CDN‐ZI glutamatergic circuit inhibition. The results indicated that, compared to the hM4D + Saline group, the hM4D + CNO group exhibited a significant increase in total sleep time (*F*_3,28_ = 5.387, *p* = 0.0047) (Figure S7D). Additionally, the number of awakening episodes per hour was significantly reduced in the hM4D + CNO group compared to the hM4D + Saline group (*F*_3,28_ = 7.993, *p* = 0.0005) (Figure S7E), and the number of NREM episodes per hour was also significantly lower in the hM4D + CNO group (*F*_3,28_ = 3.990, *p* = 0.0174) (Figure S7F), indicating that sleep fragmentation improved following chemogenetic inhibition of the CDN. Furthermore, the duration of wakefulness episodes in the hM4D + CNO group was significantly shorter than in the hM4D + Saline group (*F*_3,28_ = 6.374, *p* = 0.0020) (Figure S7G). Additionally, the duration of NREM sleep in the hM4D + CNO group was significantly longer than that in the hM4D + Saline group (*F*_3,28_ = 8.805, *p* = 0.0003) (Figure S7H), while no significant differences were observed in REM duration among the four groups (*F*_3,28_ = 0.376, *p* = 0.771) (Figure S7I). Short‐term chemogenetic inhibition of the CDN‐ZI circuit also improved sleep continuity within one day of treatment.

### 3.6 Optogenetic Inhibition of the CDN‐ZI Glutamatergic Circuit Neural Circuit Improved Sleep Disorder After TLE

Following successful modeling of pilocarpine‐induced TLE in mice, an optogenetic inhibitory virus (AAV‐CaMKIIα‐eNpHR‐EYFP) or a control virus (AAV‐CaMKIIα‐EYFP) was injected into the bilateral CDN, with optical fiber placed in the ZI region (Figure 6A). EEG/EMG was performed simultaneously, and continuous monitoring and analysis were conducted 4 weeks later. Immunohistochemistry confirmed the presence of the CDN‐ZI glutamatergic circuit after the stereotaxic injection of the optogenetic virus (Figure 6B). EEG/EMG recordings were conducted on pilocarpine‐treated mice exposed to either no light stimulation (No Light group) or yellow light stimulation (Yellow Light group) for seven consecutive days. The power spectrum and sleep stage results of mice in the eNpHR + No Light and eNpHR + Yellow Light groups (Figure 6C,D) revealed that mice in the eNpHR + No Light group exhibited fragmented NREM sleep and longer night‐awakening times (Figure 6C). Conversely, mice in the eNpHR + Yellow Light group showed improved sleep quality, increased NREM sleep duration, and reduced wake time following optogenetic inhibition of glutamatergic neurons in the CDN‐ZI glutamatergic circuit (Figure 6D).

**FIGURE 6:**
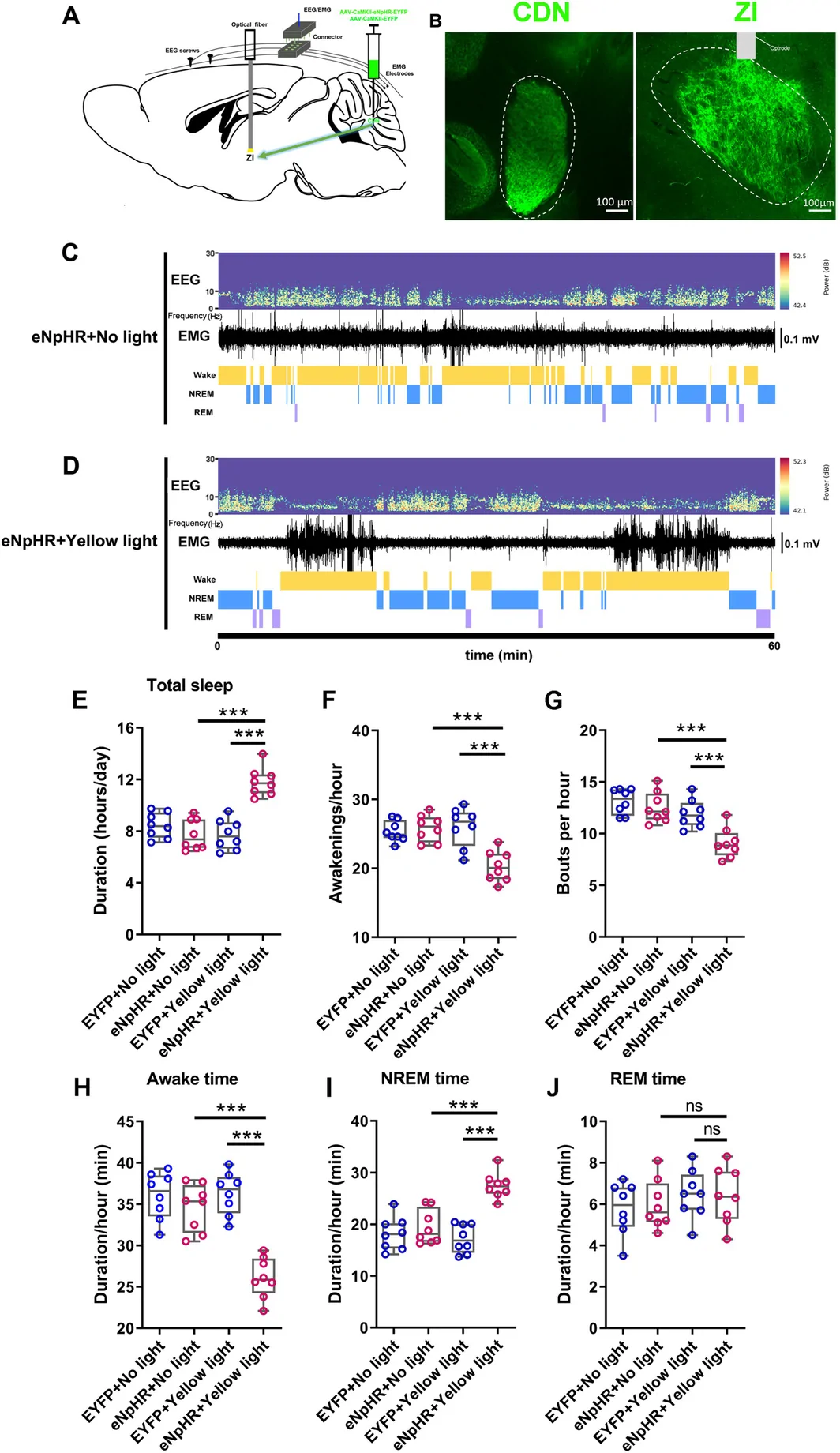
Effect of optogenetic inhibition of CDN‐ZI glutamatergic circuit on sleep disturbances after TLE. (A) Stereotaxic injection of optogenetic virus AAV2/9‐CaMKIIα‐eNpHR‐EYFP or control virus AAV2/9‐CaMKIIα‐EYFP was performed in the CDN of mice in the pilocarpine‐induced TLE model group. An optogenetic fiber was implanted in the ZI region. (B) An immunofluorescence image showing the distribution of the optogenetic virus injected into the CDN. Scale bars, 100 μm. (C, D) EEG power spectrum, electromyography (EMG), and sleep stage maps were generated for the eNpHR + No light group and eNpHR + Yellow light group. The color intensity in the EEG power spectra map indicates variations in power density. The EMG map distinguishes Wake (yellow), NREM sleep (blue), and REM sleep (purple). The EEG power spectrum is measured in square microvolts per Hertz, while the colors at the bottom of the EMG represent Wake (yellow), NREM sleep (blue), and REM sleep (purple). (E) Statistical analysis of total sleep duration of mice in EYFP + No light, eNpHR + No light, EYFP + Yellow light and eNpHR + Yellow light groups after optogenetic inhibition of CDN‐ZI glutamatergic circuit. (F) Statistical analysis of transient awakening frequency (times/h) of mice in each group after optogenetic inhibition of CDN‐ZI glutamatergic circuit. (G) Statistical analysis of Bouts per hour in each group after optogenetic inhibition of CDN‐ZI glutamatergic circuit. (H) Statistical analysis of nocturnal awakening duration (min/h) of mice in each group after optogenetic inhibition of CDN‐ZI glutamatergic circuit. (I) Statistical analysis of nocturnal NREM sleep duration (min/h) in each group of mice after optogenetic inhibition of CDN‐ZI glutamatergic circuit. (J) Statistical analysis of nocturnal REM sleep duration (min/h) of mice in each group after optogenetic inhibition of CDN‐ZI glutamatergic circuit. *n* = 8 mice/group. Data are expressed as mean ± SEM. Statistical significance was determined using one‐way ANOVA followed by Dunn's multiple comparisons test (**p < 0.01, ***p < 0.001, ns, no significant difference).

Further statistical analysis of sleep‐related indicators during awake, NREM sleep, and REM sleep phases revealed that optogenetic inhibition of the CDN‐ZI glutamatergic circuit led to a significant increase in the total sleep time in mice exposed to eNpHR + Yellow light compared with those in the eNpHR + No Light group (*F*_3,28_ = 25.17, *p <* 0.0001) (Figure 6E). The eNpHR + Yellow Light group exhibited a decrease in the number of awakenings per hour (*F*_3,28_ = 12.70, *p* < 0.0001) (Figure 6F) and bouts per hour (*F*_3,28_ = 13.66, *p* < 0.0001) (Figure 6G) during continuous sleep compared with the eNpHR + No Light group. This indicates an improvement in sleep fragmentation after the inhibition of the CDN‐ZI glutamatergic circuit. Additionally, the duration of wakefulness per hour was significantly lower in the eNpHR + Yellow Light group than in the eNpHR + No Light group (*F*_3,28_ = 26.01, *p <* 0.0001) (Figure 6H, Figure S8A). Moreover, NREM sleep duration was significantly longer in the eNpHR + Yellow Light group than in the eNpHR + No Light group (*F*_3,28_ = 20.03, *p <* 0.0001) (Figure 6I, Figure S8B), while there was no significant difference in REM sleep duration between the four groups (*F*_3,28_ = 0.57, *p* = 0.64) (Figure 6J, Figure S8C). These findings demonstrate that optogenetic inhibition of the CDN‐ZI glutamatergic circuit results in increased total sleep duration, enhanced NREM sleep quality, and reduced wake time and short wake episodes, ultimately improving sleep quality in pilocarpine‐treated mice.

To investigate the short‐term effects of optogenetic inhibition of CDN‐ZI glutamatergic circuit on sleep, we measured sleep metrics one day following CDN‐ZI glutamatergic circuit inhibition. Pilocarpine‐treated mice exposed to eNpHR + Yellow light exhibited a notable increase in total sleep time (*F*_3,28_ = 6.477, *p* = 0.0018) (Figure S8D) along with a decrease in awakenings/hour (*F*_3,28_ = 8.668, *p* = 0.0003) (Figure S8E) and NREM bouts per hour (*F*_3,28_ = 5.923, *p* = 0.0029) (Figure S8F) compared with the eNpHR + No Light group. This indicated an amelioration of sleep fragmentation in pilocarpine‐treated mice following optogenetic inhibition of the CDN. Furthermore, the eNpHR + Yellow Light group showed a significant reduction in awake duration compared with the eNpHR + No Light group (*F*_3,28_ = 5.159, *p* = 0.0058) (Figure S8G), and NREM sleep duration was significantly longer than that in the eNpHR + No Light group (*F*_3,28_ = 9.658, *p* = 0.0002) (Figure S8H). Conversely, there was no significant difference in REM sleep duration between the four groups (*F*_3,28_ = 0.8146, *p* = 0.4966) (Figure S8I). The short‐term effects of optogenetic CDN‐ZI inhibition were consistent with sustained improvements in sleep architecture.

## 4 Discussion

Patients with epilepsy frequently suffer from severe and debilitating sleep disturbances [39, 40, 41], which can, in turn, worsen seizure control [42]. However, the neurobiological underpinnings of this complex bidirectional interaction remain incompletely understood. In line with previous findings in epileptic rat models, which showed extended wakefulness and reduced NREM sleep [35, 43], our study revealed that a pilocarpine‐induced mouse model of TLE exhibited notable sleep fragmentation and decreased sleep duration, particularly during the NREM phase. By employing optogenetic and chemogenetic techniques to selectively inhibit the CDN glutamatergic neurons, we effectively reduced sleep fragmentation, increased overall sleep duration, and improved sleep quality. Our findings demonstrate the involvement of the CDN‐ZI glutamatergic circuit in the development of sleep disturbances following TLE. The inhibition of the CDN‐ZI glutamatergic circuit through optogenetics and chemogenetics led to significant improvements in sleep disturbances among mice with TLE. In conclusion, targeting the CDN‐ZI glutamatergic circuit presents a promising intervention for addressing sleep disorders associated with chronic TLE.

A growing body of evidence supports a positive correlation between sleep fragmentation and increased seizure frequency in chronic TLE. Prior research has suggested that focal seizures occurring during sleep can destabilize and fragment sleep architecture [44]. This relationship is further substantiated by a recent study showing that sleep disruption in individuals with focal drug‐resistant epilepsy promotes both ictal and interictal epileptic activity, with an increase in interictal spikes prior to arousals potentially contributing to sleep impairment [45]. Additionally, evidence from an experimental study using an animal model confirms these clinical observations. Specifically, chronic sleep fragmentation for 14 days led to increased seizure frequency and severity, along with a decrease in seizure latency in rats [46]. These findings have clinical significance, suggesting the use of sleep‐based therapeutic interventions in controlling seizures due to chronic TLE. Future prospective studies that employ objective and simultaneous measurements of seizure activity and sleep fragmentation are essential to definitively establish causality and to pave the way for chronotherapeutic strategies in epilepsy management.

In TLE animal models and patients, sleep disturbances primarily manifest as structural fragmentation of NREM sleep, characterized by increased awakenings, reduced slow‐wave activity, and decreased sleep spindle density [6]. Clinical studies indicate that frequent nocturnal awakenings in patients with TLE are often accompanied by abnormal transitions from NREM sleep to wakefulness, while changes in the proportion of REM sleep are typically less pronounced than those in NREM sleep [7]. The typical characteristic of patients with TLE is NREM sleep fragmentation. Polysomnography (PSG) studies consistently demonstrate that patients with drug‐resistant TLE exhibit reduced total sleep time, decreased sleep efficiency, an increased proportion of N1 sleep, and an elevated arousal index. In contrast, the proportion and latency of REM sleep, while variable, often do not present as major abnormalities [7]. Correlation analysis further supports this mechanism, as indicated by the correlation analysis presented in Figure S1E,F, which demonstrates that the frequency of awakenings correlates with both the frequency and duration of seizures. This finding underscores the concept that NREM fragmentation serves as a critical link between epileptic activity and sleep disturbances. Following intervention in the CDN‐ZI pathway, the continuity of NREM sleep is restored, likely due to a reduction in the arousal system's activation caused by interictal discharges. This result suggests that inhibitory strategies targeting the CDN‐ZI glutamatergic pathway may be particularly effective for addressing TLE‐related NREM maintenance disorders. In summary, the findings presented not only mechanistically elucidate the selective regulation of sleep architecture by the CDN‐ZI pathway, but also offer a biologically grounded targeted strategy for sleep management in clinical TLE patients. This strategy emphasizes the restoration of NREM stability over the generalized modulation of all sleep parameters, which may be more aligned with the pathological essence of TLE.

Our study provides mechanistic insights by confirming the existence and functional significance of the CDN‐ZI glutamatergic circuit. Previous neuroanatomical tracing studies have identified bidirectional hypothalamic‐cerebellar connections [17], and retrograde tracing from the ZI has revealed substantial projections originating from the CDN [21]. By injecting an AAV anterograde tracer virus into the CDN region, we confirmed the presence of the CDN‐ZI glutamatergic circuit. Subsequent inhibition of CDN activity through optogenetics and chemogenetics alleviated sleep fragmentation and reduced the total sleep time in pilocarpine‐treated mice with sleep disorders. This may be attributed to modulation of the glutamatergic neurotransmitter balance between excitatory glutamate and inhibitory GABA in the ZI by CDN projections. The inhibition of the CDN may lead to decreased excitatory glutamate transmission in the ZI, causing a significant increase in GABA concentration and the subsequent promotion of sleep. The outcomes included increased overall sleep time, particularly NREM sleep, decreased night awakenings, and improved sleep quality due to TLE.

The role of the cerebellum in orchestrating sleep–wake transitions is increasingly recognized. In mice, Purkinje cells show increased firing rates during the transition from NREM sleep to wakefulness, while activity in the deep cerebellar nuclei decreases [47]. Despite the crucial role of the deep thalamus in providing significant excitatory glutamatergic input to the cerebellar nuclei for maintaining arousal, this observation may seem contradictory [48, 49]. As the brain shifts from REM sleep to waking, Purkinje cells exhibit increased activity, whereas the firing rate of neurons within the deep cerebellar nucleus stays constant. In these nuclei, there are inhibitory neurons that suppress the activity of glutamatergic projection neurons [50]. This implies that the heightened functioning of Purkinje cells as the transition from sleep to wakefulness occurs could potentially boost or stabilize the output of the cerebellum by removing the inhibition on cerebellar connections to the thalamus [47]. The cerebellum is intricately connected to the neocortex in a specific and organized manner through circuits such as the corticopontocerebellar and cerebellothalamocortical circuits [51]. These connections allow for the transmission of neocortical activity to cerebellar circuits during sleep. Moreover, output from the deep cerebellar nuclei, including the CDN, projects to various thalamic nuclei (e.g., ventrolateral, ventromedial, and centrolateral nuclei), thereby influencing multiple thalamocortical networks in a target‐dependent manner [52]. Our findings add a crucial piece to this puzzle by identifying the ZI as a key downstream mediator of cerebellar influence on sleep.

Despite the critical role of the hypothalamus and brainstem in sleep–wake regulation, the cerebellum's function has remained relatively underexplored [53]. However, human imaging studies have shown that cerebellar activity covaries with neocortical activity across different sleep stages [54], and electrophysiological recordings have demonstrated that firing patterns in cerebellar nuclei and Purkinje cells are modulated by the neocortical sleep cycle [54, 55, 56]. Cerebellar dysfunction can have profound effects on sleep–wake regulation, contributing to a spectrum of sleep disorders, including insomnia, excessive daytime sleepiness, REM behavior disorder, and sleep apnea [8, 54, 57]. For example, patients with spinocerebellar ataxias show elevated daytime drowsiness and parasomnias [57], while those with primary sleep disorders often exhibit reduced cerebellar volume and dysfunction [58, 59].

While our study offers compelling evidence for the role of the CDN‐ZI circuit in TLE‐related sleep disturbances, several limitations warrant consideration. First, our experiments were conducted exclusively in male mice, and it remains to be determined whether these findings are generalizable to females. Second, although our results demonstrate a clear improvement in sleep, we did not observe a significant reduction in seizure frequency or duration following CDN or CDN‐ZI inhibition. This suggests that the sleep‐promoting effects of this pathway are likely independent of its direct modulation of epileptogenesis. Future studies should explore whether chronic, rather than acute, manipulation of this circuit over extended periods might confer both sleep and seizure benefits. Third, the specificity of this pathway should be validated across different epilepsy models to ascertain the most broadly effective therapeutic strategies. In conclusion, our study establishes the CDN‐ZI glutamatergic pathway as a key regulator of sleep in the context of chronic TLE. Targeted inhibition of this circuit represents a novel and promising therapeutic avenue for alleviating the debilitating sleep disorders that are a hallmark of this condition.

## Author Contributions

**Liang Xia:** writing – review and editing, writing – original draft, visualization, methodology, investigation. **Xuan Wang:** writing – original draft, visualization, validation, software, methodology, investigation. **Yingchi Zhang:** writing – review and editing, supervision, resources, project administration, funding acquisition, data curation, conceptualization. **Jianmin Hao:** writing – review and editing, supervision, resources, data curation. **Dan Mi:** resources, investigation, software, formal analysis. **Changgeng Song:** writing – review and editing, conceptualization. **Wen Jiang:** supervision, resources, project administration, funding acquisition, data curation.

## Funding

This work was supported by the National Natural Science Foundation of China (No. 82471471), Shaanxi Province Special Support Program for Leading Talents in Scientific and Technological Innovation (No. tzjhjw).

## Ethics Statement

All procedures carried out on the animals were in accordance with the guidelines set by the Animal Use and Laboratory Animal Management Ethics Committee of Air Force Medical University and were approved by the Ethics Committee of Xijing Hospital (XJ‐20231018‐F1).

## Conflicts of Interest

The authors declare no conflicts of interest.

## Supporting information


**Figure S1:** The frequency and duration of spontaneous recurrent seizures (SRS) are correlated with the frequency of awakenings in a mouse model of TLE.

**Figure S2:** Effect of chemogenetic inhibition of CDN glutamatergic neurons on epilepsy after TLE.

**Figure S3:** Effect of optogenetic inhibition of CDN glutamatergic neurons on epilepsy after TLE.

**Figure S4:** Pilocarpine‐treated mice exhibit significant sleep disturbances.

**Figure S5:** Effects of chemogenetic inhibition of CDN glutamatergic neurons on sleep disorders.

**Figure S6:** Effects of optogenetic inhibition of CDN glutamatergic neurons on sleep disorders.

**Figure S7:** Effects of chemogenetic inhibition of CDN‐ZI glutamatergic neurons on sleep disorders.

**Figure S8:** Effects of optogenetic inhibition of CDN‐ZI glutamatergic neurons on sleep disorders.

## Data Availability

Data will be made available on request.
